# CD57+ T-cells are a subpopulation of T-follicular helper cells in nodular lymphocyte predominant Hodgkin lymphoma

**DOI:** 10.1186/s40164-015-0022-1

**Published:** 2015-09-15

**Authors:** Ahmad Sattarzadeh, Arjan Diepstra, Bea Rutgers, Anke van den Berg, Lydia Visser

**Affiliations:** Department of Pathology and Medical Biology, University of Groningen, University Medical Center Groningen, Groningen, The Netherlands

**Keywords:** Nodular predominant Hodgkin lymphoma, CD57+ T cells, TFH cells

## Abstract

**Background:**

Nodular lymphocyte predominant Hodgkin lymphoma (NLPHL) is characterized by lymphocyte-predominant (LP) cells in a background of CD4+ CD57+ T-cells. These cells are normally present in the germinal center of lymphoid tissues. The cells rosetting LP cells are described to be PD-1 and BCL-6 positive, which are markers of T-follicular helper cells. This study was designed to address the question: are the CD57+ T cells in germinal centers of tonsil and NLPHL TFH cells?

**Results:**

Immunohistochemistry was performed on tonsil and NLPHL. For tonsil, cells per germinal center and for NLPHL, the area around LP cells was counted. Cells rosetting LP cells were also determined. In addition, flowcytometry was performed on cell suspensions. Cells directly rosetting LP cells are positive for CD57 and/or for two markers of T-follicular helper (TFH) cells, PD-1 and BCL-6. We show that in both tonsil and NLPHL more than 90 % of CD57+ T-cells are also positive for PD-1, whereas roughly half of the PD-1+ T-cells are CD57+. CD57+ cells co-express BCL-6 in tonsil and in the rosetting cells of NLPHL.

**Conclusions:**

We conclude that CD57+ T-cells are TFH cells and form a subpopulation of TFH cells in tonsils and NLPHL.

## Background

Nodular lymphocyte predominant Hodgkin lymphoma (NLPHL) accounts for 5 % of all Hodgkin lymphoma cases. Lymphocyte-predominant (LP) cells, the neoplastic cells of NLPHL, compose only 1 % of the total cell population and reside in a background of lymphoid cells. One of the prominent cell types in NLPHL are the CD4+ CD57+ T-cells. These CD57+ T-cells are normally present in the germinal centers of tonsil and form about 5–8 % of all cells. In NLPHL, CD4+ CD57+ T-cells form 20–40 % of the total cell population and they are present mostly around the LP cells [1]. Another CD4+ T-cell subpopulation in the germinal centers of tonsils are the T-follicular helper (TFH) cells. TFH cells are critical cells for B-cell maturation [2]. They can be distinguished from other T helper subpopulations by co-expression of the TFH cell associated transcription factors c-Maf and BCL-6 in addition to expression of PD-1, CXCR5, CXCL13 and ICOS [3]. In NLPHL, BCL-6+ CD57+ T-cells [4] and c-Maf+ CD57+ T-cells [5] rosetting the LP cells have been reported. Expression of known TFH cells markers such as PD-1 has also been observed on rosetting cells in NLPHL and PD-1 outperforms CD57 as an additional diagnostic tool in the diagnosis of NLPHL [6, 7]. This study was designed to determine whether CD57+ T-cells in NLPHL are TFH cells and if they are similar to the TFH cells in normal tonsil tissue.

## Materials and methods

Paraffin blocks of 6 tonsils (chronic tonsillitis in young patients) and lymph nodes of 8 NLPHL patients were used for staining. For flowcytometry, cell suspensions stored in liquid nitrogen of 7 tonsils and the same 8 NLPHL lymph nodes were used. The study protocol was consistent with international ethical and professional guidelines (the Declaration of Helsinki and the International Conference on Harmonization Guidelines for Good Clinical Practice).

For immunohistochemistry and immunofluorescence stainings of PD-1 (1:100, Acris, Herford, Germany) in combination with CD57 (1:50, Monosan, Uden, The Netherlands) antigen retrieval was performed with 0.1 M TRIS/HCl pH9 in a microwave. For BCL-6 (1:20, BD biosciences, San José, CA, USA) in combination with CD57 and CD20 (1:200, Dako, Glostrup, Denmark) antigen retrieval in a high pressure cooker in 1 mM EDTA pH8 was performed. In order to count the CD57+ and PD-1+ cells in tonsils, all PD-1+, CD57+ and double positive cells in at least five germinal centers were counted in each sample. In NLPHL, cells present around LP cells in 10 areas with the magnification of 200× were counted for CD57+, PD-1+ and double positive cells. In addition, in each case the rosetting cells of at least 15 LP cells were counted with the magnification of 400×. Standard laboratory procedures were followed for all stainings, including appropriate positive and negative controls.

For flowcytometry 0.5 × 10^6^ cells were incubated with anti-CD4, anti-PD-1, and anti-CD57 (BD biosciences). Cells were acquired on a Calibur (BD biosciences) and analyzed with Winlist software.

A Mann–Whitney test was applied to determine significant differences between groups.

### Results

#### Immunohistochemistry and immunofluorescence results

**Fig. 1:**
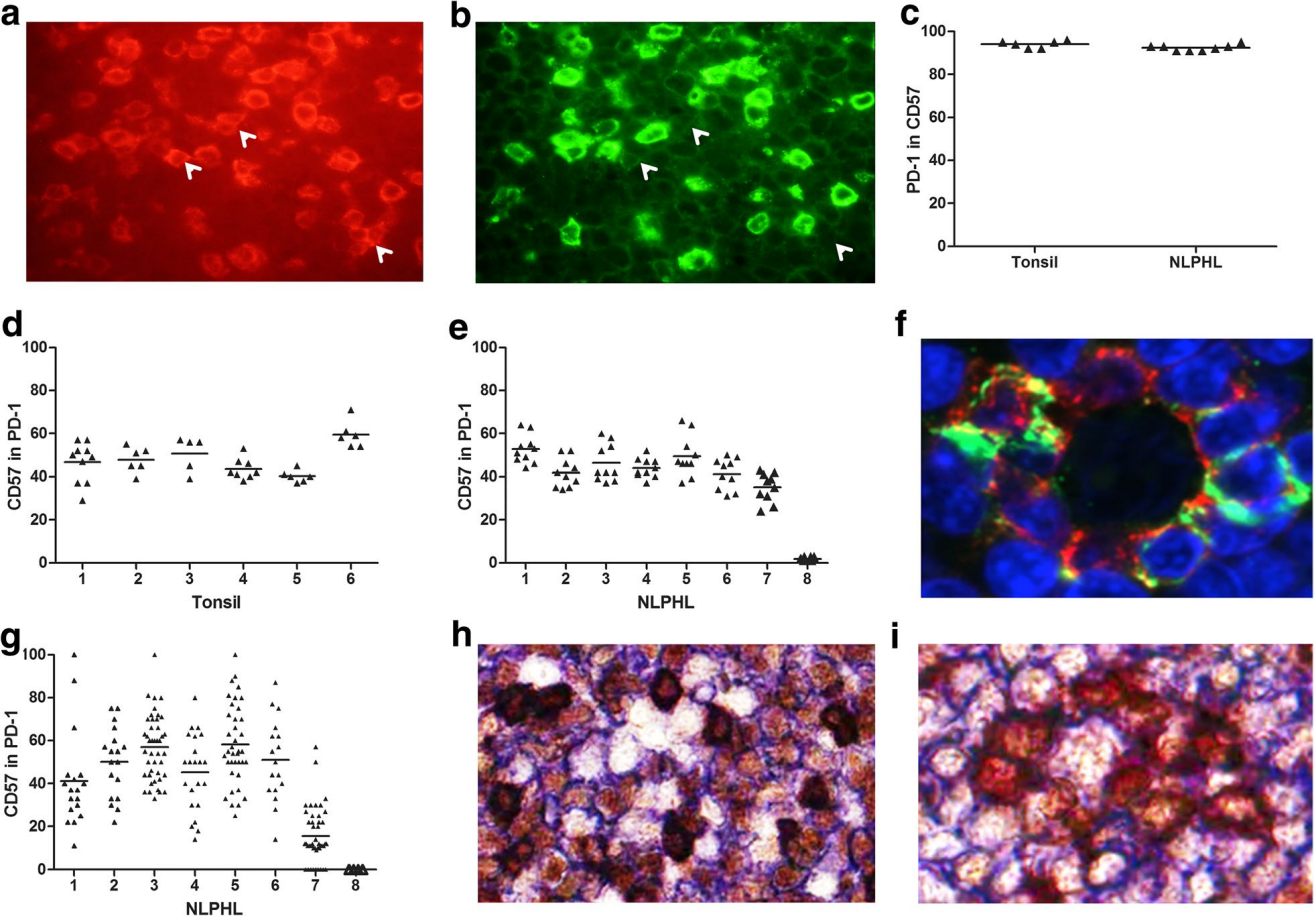
Immunohistochemistry results of tonsil and NLPHL. **a** PD-1 (*red*) and **b** CD57 (*green*) fluorescent staining of tonsil. Shown is exactly the same area of a germinal center (×400) with different filters. *Arrows* indicate some of the PD-1+ cells which are CD57−. **c** Percentage of PD-1+ cells in the CD57+ T-cell population in tonsil and NLPHL; 94 and 92 % of the CD57+ cells express PD-1 in tonsil and NLPHL, respectively. **d** Percentage of CD57+ cells in the PD-1+ population in tonsil; an average of 48 % of PD-1+ cells are CD57+. **e** Percentage of CD57+ cells in PD-1+ population in NLPHL, an average of 39 % of PD-1+ cells are CD57+. **f** PD-1(*red*) and CD57 (*green*) staining of NLPHL (×800). The nucleus is counter stained with DAPI (*blue*). In the center an LP cell surrounded by PD-1+ cells, three of which are also positive for CD57 (*green yellow*) is shown. **g** Percentage of CD57+ cells in PD-1+ rosetting cells in NLPHL, the range of PD-1+ LP rosetting cells that are CD57+ varies from 0 to 58 %. **h** CD57 (*dark red* rim), BCL-6 (*brown* nucleus), CD20 (*blue*) staining in tonsil (×400). CD57+ cells are also BCL-6+. Some of the CD20+ cells also express BCL-6. Part of the cells are single positive for BCL-6. **i** CD57 (*dark red* rim), BCL-6 (*brown* nucleus), CD20 (*blue*) staining in NLPHL: (×600). An LP cell (CD20+ and BCL-6+) is surrounded with two types of cells: BCL-6 and CD57 double positive cells and BCL-6 single positive cells

Immunofluorescence staining of PD-1 in combination with CD57 showed that almost all CD57+ cells in the germinal centers were also PD-1 positive, while part of the PD-1+ cells were negative for CD57 (Fig. 1a, b). Counting of all available (5–10) germinal centers in each tonsil and 10 LP cell rich areas in NLPHL for PD-1 and CD57 positivity indicated that 94 and 92 % of the CD57+ T-cells expressed PD-1 in both tonsil and NLPHL (Fig. 1c). Of the PD-1+ T-cells, 48 and 39 % were CD57+ in tonsil and NLPHL, respectively (Fig. 1d, e).

We next counted the cells immediately rosetting LP cells for PD-1 and CD57 expression (17–45 LP cells per case). We observed two populations of cells, single PD-1 expressing cells and cells expressing both PD-1 and CD57 (Fig. 1f). Virtually all rosetting cells were PD-1 positive, while the percentage of PD-1+ CD57+ varied between 0 and 58 % (Fig. 1g).

Immunohistochemical staining for BCL-6, CD57 and CD20 indicated that almost all CD57+ T-cells in the germinal center of tonsils were BCL-6 positive (Fig. 1h). In addition, we observed multiple BCL-6 single positive T-cells. In NLPHL we saw a similar pattern, with all CD57+ T-cells within the area around the LP cells as well as the LP rosetting cells being BCL-6 positive (Fig. 1i). In addition, we also observed a substantial number of single BCL-6+ T-cells in the areas of the LP cells as well as directly rosetting around the LP cells.

### Flowcytometry results

**Fig. 2:**
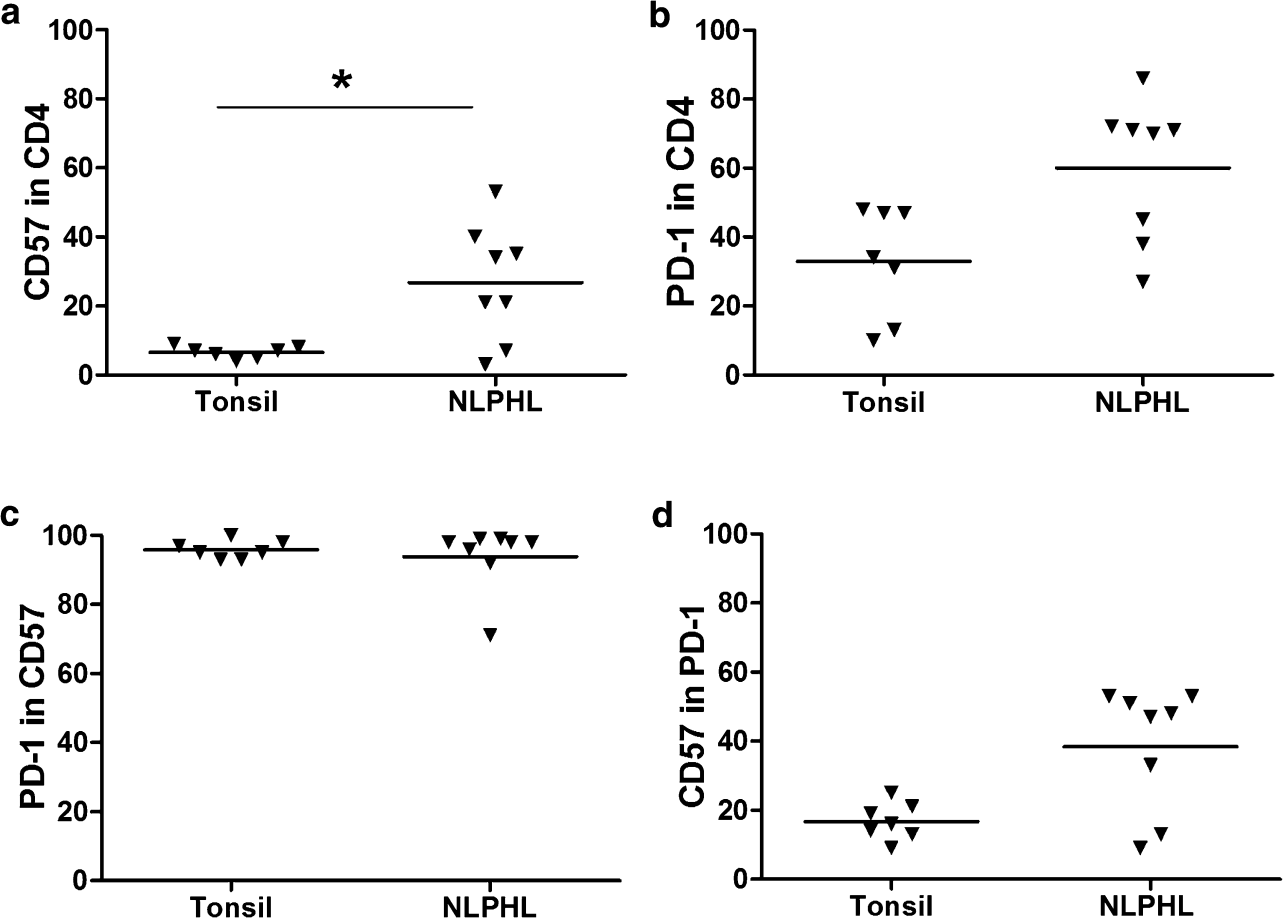
Flowcytometry results for tonsil and NLPHL cell suspensions. **a** Percentage of CD57+ T-cells in CD4+ T-cells in tonsil and NLPHL, the average was 5 and 25 %, respectively (*p < 0.05). **b** Percentage of PD-1+ cells in CD4+ T-cells in tonsil and NLPHL, the average was 33 and 60 %, respectively. **c** PD-1+ cells in the CD57+ T-cell population in tonsil and NLPHL was 96 and 94 %, respectively. **d** Percentage of CD57+ cells in the PD-1+ T-cell population in tonsil and in NLPHL was 17 and 38 %, respectively

In both tonsil and NLPHL suspensions we gated on the CD4+ T-cells and we determined the percentages of PD-1 and CD57 positive cells in the whole cell suspension. The percentage of CD57+ cells was 5 % on average in tonsil, and 25 % on average in NLPHL, and was the only significant difference (p = .042) (Fig. 2a). A higher percentage of PD-1+ cells was observed in NLPHL compared to tonsil, 60 vs 33 % (Fig. 2b). Almost all CD57+ T-cells were positive for PD-1, i.e. 96 % in tonsil and 94 % in NLPHL (Fig. 2c). In contrast, only part of the PD-1+ T-cells were positive for CD57, i.e. 17 % in tonsil and 38 % in NLPHL.

## Discussion

To establish whether CD57+ T-cells in NLPHL are TFH cells, we analyzed co-expression with PD-1 and BCL-6. We showed that more than 90 % of CD57+ cells in the germinal center of tonsil and the tumor areas in NLPHL are PD-1 positive. These CD57+ cells are a subpopulation of the PD-1+ population as they represent an average of 48 % of the PD-1+ cells in germinal centers in tonsil and 39 % in the tumor areas in NLPHL. There is a large variation in the percentages of CD57+ cells in the PD-1 subpopulation in NLPHL, this could be due to the differences in morphological patterns of the cases. It has been described that in diffuse areas of the tumor less PD-1 and CD57 positivity is seen [7]. Of the 8 cases tested 6 (cases 1–6) were classic nodular, case 7 was a case with T-cell rich nodules and case 8 was a case with prominent extra nodular LP cells that was CD57 negative. The large variation in percentage within the individual cases cannot be found in differences in diffuse and nodular areas. The lymphocytes rosetting the LP cells are PD-1 positive as reported earlier [6] and CD57+ cells form a subpopulation of the rosetting cells in most patients. Flowcytometry confirmed that in both tonsil and NLPHL more than 90 % of CD57+ T-cells express PD-1. In NLPHL, the percentage of CD57+ cells in the PD-1 population is comparable with staining results (39 and 38 %). In tonsil, the percentage of CD57+ cells in the PD-1 population is much lower as determined by flowcytometry (48 vs 17 %). The explanation for this discrepancy might be that PD-1+ T-cells are also present outside the germinal centers in tonsil [8], while CD57+ T-cells are only found in the germinal centers [9]. For the immunostaining we specifically counted the germinal centers in tonsils, which has resulted in higher percentages.

CD57+ cells in tonsil as well as in the areas around the tumor cells and the LP rosetting cells express BCL-6 in concurrence with previous studies about the presence of BCL-6+ LP rosetting T-cells [4, 10]. A significant increase was observed by flowcytometry in terms of CD57 percentages in NLPHL cases compared to tonsil, which has been reported previously in flowcytometry [1]. Co-expression of PD-1 as a known marker of TFH cells with the TFH cell associated transcription factor BCL-6 suggests that these CD57+ T-cells most likely are TFH cells. This is also supported by the common expression of c-Maf, which is another transcription factor of TFH cells, in LP rosetting cells [5].

Although TFH cells are important in providing help to B-cells especially in the area of antibody responses [2], CD57+ CD4 cells have been described as cells that are not able to proliferate due to chronic antigen exposure [11]. These cells produce interferon-γ but are not able to produce interleukin-2 [10, 12] and have the ability to suppress activation of conventional CD4 cells [12]. Their function in NLPHL is not clear, but CD57+ TFH cells could play a role in immune suppression of the microenvironment. The presence of PD-1+ cells has recently gained interest as a potential therapeutic target in cancer. In classical Hodgkin lymphoma blocking of PD-1 by nivolumab was very successful [13]. The presence of PD-1+ T cells seems less predictive of the success of blocking PD-1/PD-L1 however as the expression of PD-L1 by tumor cells, and PD-L1 expression in NLPHL is found in few cases [14].

In conclusion, we show that LP cells are rosetted by PD-1+ BCL-6+ double and PD-1+ CD57+ BCL-6+ triple positive T-cells. In addition, we show a remarkable increase in the population of CD4+ PD-1+ and CD4+ PD-1+ CD57+ T-cells in NLPHL. CD57 positive CD4 cells are a subpopulation of TFH cells with a potential role in the pathogenesis of NLPHL.
